# Evaluation of hepatobiliary involvement in children with cystic fibrosis: a retrospective single-center study

**DOI:** 10.1186/s12876-026-05186-6

**Published:** 2026-08-05

**Authors:** Ahmet Uzger, Ahmet Rauf Goktepe, Pelin Asfuroğlu, Yasin Sahin

**Affiliations:** 1 Department of Pediatric Gastroenterology, Gaziantep City Hospital, Gaziantep, Gaziantep, 27560 Turkey; 2 Clinic of Pediatric Pulmonology, Gaziantep City Hospital, Gaziantep, Gaziantep, 27560 Turkey; 3 Faculty of Medicine, Gaziantep Islam Science and Technology University, Gaziantep, Gaziantep, 27560 Turkey

**Keywords:** Children, Cystic fibrosis, Hepatobiliary involvement, Ultrasound

## Abstract

**Background:**

It is difficult to know the actual prevalence of liver disease in patients with cystic fibrosis (CF) because of the lack of an accepted definition of cystic fibrosis-associated liver disease (CFALD), the fact that most patients are asymptomatic, and the lack of specific noninvasive diagnostic tests. This study aimed to investigate the frequency and characteristics of liver involvement in patients diagnosed with CF.

**Methods:**

This retrospective study was conducted from January 2024 to January 2025. Pediatric patients aged 1 − 18 years diagnosed with CF were included. All patients were evaluated for clinical and laboratory findings related to liver disease. Only patients with liver function test results and/or ultrasonography (US) findings were included in the study regardless of symptoms. Children with comorbid diseases other than CF, such as chronic liver disease, kidney disease, cerebral palsy, or type 1 diabetes mellitus, were excluded.

**Results:**

Sixty patients were included in the study. The mean age was 7.17 ± 5.69 (0.4–18.0) years. 31 (51.7%) of the patients were male. The mean ALT values of patients were 29.91 ± 23.52 IU/L. Ultrasound imaging was not performed in 13 patients. Among the 47 patients who underwent ultrasonography, 13 had abnormal findings and 34 had normal findings. Ten patients (16.6%) had abnormal liver function tests during follow-up. Five patients had hepatomegaly, six had a heterogeneous granular appearance, and two had hepatosteatosis. Three patients with a heterogeneous appearance on ultrasound had elevated serum transaminase levels. All patients with hepatomegaly, except one, had normal serum transaminase levels. Furthermore, one patient underwent a liver biopsy, and the biopsy findings were consistent with cirrhosis.

**Conclusions:**

Hepatobiliary involvement was detected in 18 (30.0%) patients. Elevated transaminase levels were not significantly associated with abnormal ultrasonographic findings. Our findings support regular liver disease screening in children with CF, including abdominal ultrasonography, to facilitate early recognition and management.

## Background

Cystic fibrosis (CF) is a common disease among Caucasians that shortens life expectancy. The global prevalence of CF is estimated to range from 1 in 3,000 to 1 in 7,000, although it varies by country [1].

Structural and functional abnormalities of the Cystic Fibrosis Transmembrane conductance regulator (CFTR) gene lead to the production of thick, sticky mucus, resulting in dysfunction of organs such as the lungs, pancreas, liver, intestines, sweat glands, and epididymis. The lungs are the most affected organs in CF, and the resulting respiratory failure is the most common cause of mortality [2, 3]. Cystic Fibrosis-Associated Liver Disease (CFALD) develops in early childhood and is the third most common cause of mortality in CF patients, accounting for 3.2% of total mortality [4, 5]. Determining the true prevalence of liver disease in patients with CF is challenging because of the lack of a universally accepted definition of CFALD, the fact that most patients are asymptomatic, and the absence of specific noninvasive diagnostic tests [2]. CFALD has been reported in approximately 30% of patients with CF [6]. During the course of the disease, transient transaminase elevations may be detected, especially during pulmonary exacerbations or due to medication use. Hepatosteatosis is a common liver finding in CF and its prevalence has been reported to be between 23 and 70% [2]. Liver involvement is usually subclinical, and patients may remain asymptomatic until significant liver damage has developed.

Regular screening is critical for early detection of liver disease in patients with CF. Current guidelines recommend annual screening for liver disease in patients with CF [7–9]. This study aimed to investigate the frequency and characteristics of liver involvement in patients diagnosed with CF.

## Methods

**Fig. 1:**
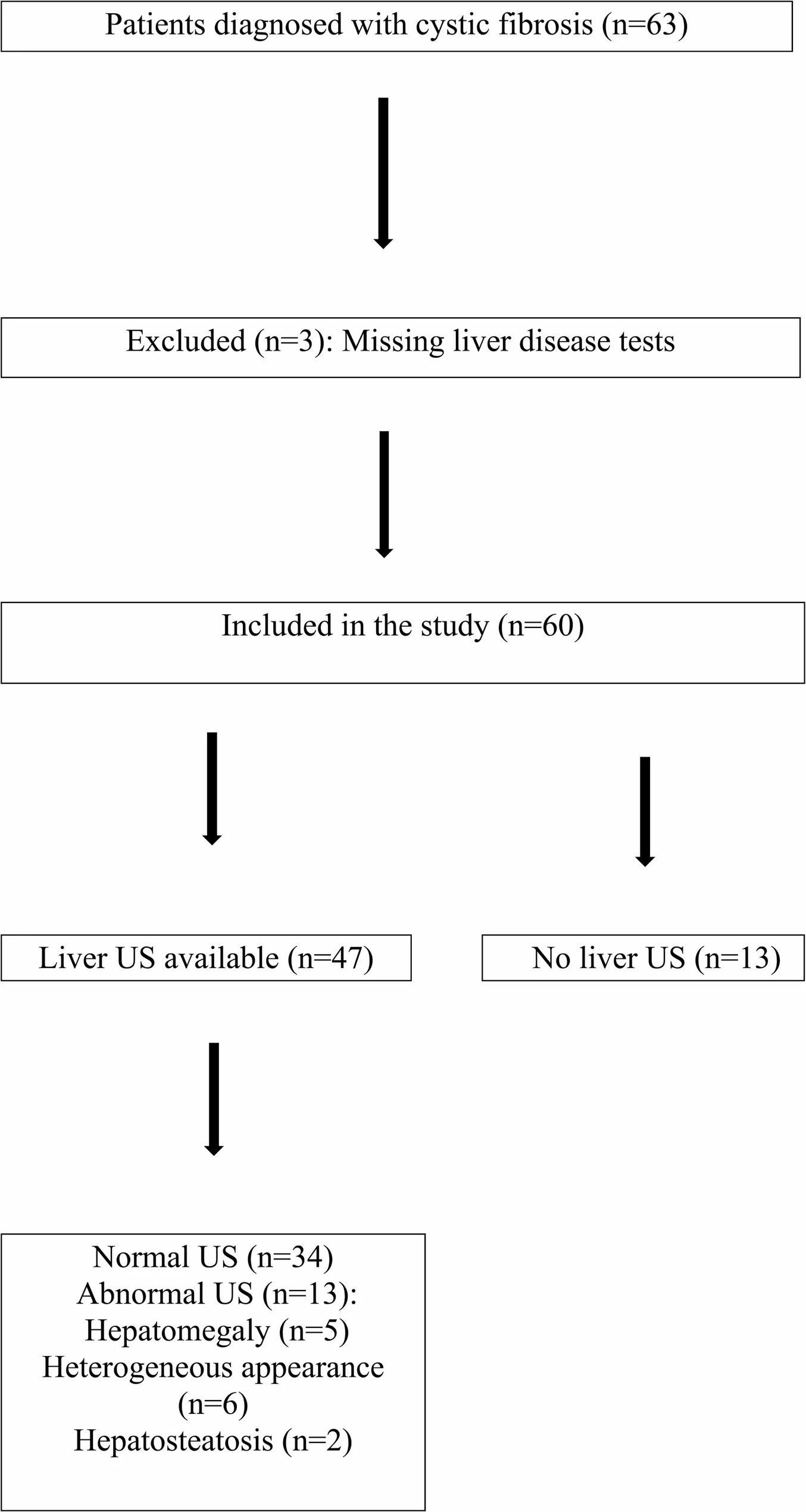
Patient inclusion and exclusion flowchart. US: Ultrasonography

This retrospective study was conducted from January 2024 to January 2025. A total of 60 patients aged 1–18 years who attended the Pediatric Gastroenterology and Pediatric Pulmonology outpatient clinics with a diagnosis of CF were included in the study (Fig. 1). The diagnosis of CF was established according to generally accepted diagnostic criteria [10].

Because the study was retrospective, all pediatric patients diagnosed with CF who had liver function test results available in their medical records were included in the study. Patients were referred to the pediatric gastroenterology department by a pediatric pulmonologist, but some had never attended the clinic. Therefore, regardless of the presence or absence of symptoms, only patients with available liver function test results and/or ultrasonography (US) findings were included in the study. Consequently, these patients had not undergone routine liver disease screening.

Children with comorbid chronic diseases other than CF, such as chronic liver disease, kidney disease, cerebral palsy, or type 1 diabetes mellitus, were excluded. Three patients with missing liver function test results were also excluded. Additionally, patients with transient enzyme elevations due to infections, medications, or nutritional support were excluded from the study; only patients with persistent enzyme elevations documented on at least two separate occasions were included.

Patients with hepatobiliary involvement were followed in the pediatric gastroenterology outpatient clinic for one year. A standardized retrospective data collection form was developed for this study to systematically extract demographic, clinical, laboratory, imaging, treatment, and follow-up data from electronic medical records.

Liver ultrasonography was performed using a 3–5 MHz convex transducer, which provided adequate penetration and parenchymal resolution [11]. Ultrasonographic examinations were performed by radiologists who were unaware of the patients’ clinical data.

The cut-off value for serum ALT was 40 U/L. Values above this threshold were considered abnormal. Patients with elevated liver enzyme levels on at least two separate occasions obtained at different time points were considered to have hepatobiliary involvement.

In the evaluation of hepatobiliary involvement, the presence of one or more of the following criteria was considered sufficient according to the CF Foundation consensus recommendations [8]. CFALD is defined as the presence of one or more of the following: [1] nodular liver, advanced fibrosis (F4), multilobular cirrhosis with or without portal hypertension, or noncirrhotic portal hypertension; [2] hepatomegaly, liver fibrosis (< F4), increased liver stiffness by elastography (< F4), hepatic steatosis, focal biliary cirrhosis, cholestasis, persistently elevated serum liver function tests (any value above upper limit of normal), abnormal liver imaging, cholelithiasis, sclerosing cholangitis, or hepatolithiasis.

This study was conducted in accordance with the principles of the Declaration of Helsinki. This study was approved by the Gaziantep City Hospital Non-Interventional Research Ethics Committee (Approval No. 114/2024; January 15, 2024). The Institutional Review Board waived the requirement for informed consent because the research involved analysis of retrospective data.

This study was designed and reported in accordance with the STROBE (Strengthening the Reporting of Observational Studies in Epidemiology) guidelines to ensure transparency and completeness in reporting observational research. 

### Statistical analysis

Descriptive statistics were presented as numbers, percentages, mean ± standard deviation (SD), or median (interquartile range(IQR)), as appropriate. Continuous variables were compared using the Student’s t-test for normally distributed data and the Mann–Whitney U test for non-normally distributed data. The association between abnormal ALT levels (> 40 U/L) and abnormal liver ultrasonography findings was assessed using Fisher’s exact test. The strength of the association was expressed as an odds ratio (OR). A two-sided p value of < 0.05 was considered statistically significant.

## Results

Sixty patients with CF were included in the study. The mean age was 7.17 ± 5.69 (range, 0.4 − 18.0) years. The median age at the diagnosis of CF was 0.73 years (0,33 − 5,75). Thirty-one (51.7%) patients were male. All patients were evaluated for clinical and laboratory findings related to liver disease. The mean AST and ALT levels were 41.62 ± 24.96 IU/L and 29.91 ± 23.52 IU/L, respectively. The total bilirubin and direct bilirubin levels were 0.46 ± 0.48 mg/dL and 0.099 ± 0.09 mg/dL, respectively. The albumin value of patients was 41.36 ± 6.93 g/L (Table 1).

**Table 1:** Demographic features and the laboratory characteristics of the patients

Age of the patients	7.17±5.69
Sex (m/f)	31/29
Age of the patients at the time of diagnosis of CF (years)*	0.73(0.33-5.75)
Hemoglobin value (g/dL)	12.65±1.78
AST values (U/L)	41.62±24.96
ALT values (U/L)	29.91±23.52
Total bilirubin levels (mg/dL)	0.46±0.48
Direct bilirubin levels (mg/dL)	0.099±0.09
The albumin value (g/L)	41.36±6.93
GGT levels (U/L)	22.05±25.37

*ALT* Alanine aminotransferase, *AST* Aspartate aminotransferase, *CF* Cystic fibrosis, *GGT* Gamma-glutamyl transferase

*Data are presented as median (IQR)

Ultrasonography was performed in 47 patients. US findings were abnormal in 13 patients (five with hepatomegaly, six with a heterogeneous granular appearance, and two with hepatosteatosis), while 34 patients had normal findings. Five of the 13 patients who had abnormal US findings had abnormal liver function tests during follow-up (Table 2). No biliary tract abnormalities or gallstones were observed in any patient. Additionally, no splenic abnormalities were identified in any patient.

**Table 2:** Clinical characteristics of patients with hepatobiliary involvement according to liver ultrasonography findings

US: normal				
Case	Sex	Age (years)	ALT level (U/L)	
1	f	15.3	45	
2	m	0.8	49	
3	m	16.5	125	
4	f	0.83	89	

US: Hepatomegaly				
Case	Sex	Age (years)	ALT level	
1	m	16.5	43	
2	m	7.8	27	
3	f	1.1	21	
4	m	15.1	18	
5	f	5.3	12	

US: Heterogeneity				
Case	Sex	Age (years)	ALT level	
1	m	10.3	55	
2	m	18	84	
3	m	0.7	113	
4	m	4	14	
5	f	1.9	29	
6	m	0.92	15	

US: Hepatosteatosis				
Case	Sex	Age (years)	ALT level	grade
1	m	14	32	1
2	m	16.5	43	2

US: not available				
Case	Sex	Age (years)	ALT level	
1	f	4.5	63	

*ALT* Alanine aminotransferase, *US* Ultrasonography

Five of the 13 patients with abnormal liver ultrasonography findings had abnormal liver function tests during follow-up (Table 2). Ten of the 60 patients (16.6%) had abnormal liver function tests during follow-up (Table 2). None exhibited hypoalbuminemia, hyperbilirubinemia, or prolonged prothrombin time. Three patients with a heterogeneous appearance on ultrasonography had elevated serum transaminase levels. All patients with hepatomegaly, except one, had normal serum transaminase levels. One patient with elevated transaminase levels did not undergo liver ultrasonography because the patient did not attend follow-up visits. Only one patient underwent a liver biopsy because elevated liver enzyme levels had persisted for six months, and the biopsy findings were consistent with cirrhosis.

There was no statistically significant association between abnormal ALT levels (> 40 U/L) and abnormal liver ultrasonography findings (Fisher’s exact test, OR = 4.69, *p* = 0.091; Table 3). Although patients with elevated ALT levels were more likely to have abnormal ultrasonographic findings, the difference did not reach statistical significance.

**Table 3:** Association between serum ALT levels and liver ultrasonography findings

Variable	Normal US	Abnormal US	OR (95% CI)	*p* value*
ALT≤40 (U/L)	30 (88.2%)	8 (61.5%)	Reference	
ALT>40 (U/L)	4 (11.8%)	5 (38.5%)	4.69	0.091

*ALT* Alanine aminotransferase, *US* Ultrasonography

Odds Ratio (OR): 4.69

*Fisher's exact test (two-sided)

Ursodeoxycholic acid (UDCA) treatment was initiated at a dose of 20 mg/kg/day in 18 patients after hepatobiliary involvement was detected. Patients with hepatobiliary involvement were re-evaluated one month after diagnosis, and if their clinical and laboratory findings remained stable, follow-up visits were scheduled every 2–3 months. Following initiation of UDCA treatment, ALT levels increased in two patients with elevated baseline ALT levels and in one patient with hepatomegaly who had normal baseline ALT levels. ALT levels decreased in eight patients. ALT levels remained stable in seven patients whose baseline ALT levels were below 40 U/L. During the available follow-up period, no changes were observed in liver ultrasonography findings or clinical outcomes. Nutritional support was provided to patients with hepatic steatosis. Among patients receiving UDCA treatment, no statistically significant difference was observed in ALT levels before and after treatment (*p* = 0.415; Table 4).

**Table 4:** Pre- and post-treatment comparison of ALT levels in patients receiving UDCA

	Pre-treatment	Post-treatment	*p* value
ALT levels (U/L)	50.22±34.52	40.77±34.17	0.415

*ALT* Alanine aminotransferase, *UDCA* Ursodeoxycholic acid

## Discussion

In our study, we evaluated the prevalence, clinical features, elevated transaminase levels, and ultrasonographic findings of CFALD to emphasize the importance of early diagnosis and management strategies. Hepatobiliary involvement was detected in 18 (30.0%) patients. The reported prevalence of hepatobiliary involvement in cystic fibrosis varies considerably across studies, largely because of differences in diagnostic definitions and inclusion criteria. Therefore, the prevalence observed in our study should be interpreted within the context of the Cystic Fibrosis Foundation consensus criteria used for patient classification. Ultrasound findings were abnormal in 13 patients (five with hepatomegaly, six with a heterogeneous granular appearance, and two with hepatosteatosis), while 34 patients had normal findings. Five of the 13 patients with abnormal US findings had abnormal liver function tests during follow-up. 10 of the 60 patients (16.6%) had abnormal liver function tests during their follow-up. Only one of the patients underwent a liver biopsy because of persistently elevated liver enzyme levels for six months, and the biopsy findings were consistent with cirrhosis.

Cystic fibrosis is a multisystem disease caused by mutations in the CFTR gene. Liver involvement is a significant cause of morbidity and mortality, particularly in pediatric patients [12, 13]. Focal biliary cirrhosis is the pathognomonic liver disease observed in patients with CF [14]. Over time, localized biliary cirrhosis may progress to multilobular cirrhosis in some patients, leading to portal hypertension (HT) and its associated complications [7]. Portal HT develops in 5–10% of patients. Portal HT and secondary variceal bleeding are among the most serious complications of CFALD. Patients who develop complications of treatment-resistant portal HT and/or end-stage liver failure should be evaluated for liver transplantation [15]. In our study, only one patient underwent liver biopsy and the biopsy findings were compatible with cirrhosis and the patient was referred to a liver transplant center.

Determining the true prevalence of liver disease in patients with CF is challenging because of the lack of a universally accepted definition of CFALD, the fact that most patients are asymptomatic, and the absence of specific noninvasive diagnostic tests [16]. Although CFALD is generally asymptomatic in the early stages, elevated transaminase levels and ultrasonographic abnormalities are common and require regular follow-up. The prevalence of CFALD has been reported to range from 10% to 40% [6, 17, 18]. Consistent with previous reports, hepatobiliary involvement was detected in 18 (30.0%) patients in our study.

In a prospective study conducted by Woodruff et al. [19], 298 patients with CF were followed up until the age of 21, and it was found that 93% of the patients had at least one elevation in ALT levels during the follow up period. The study also found that elevated ALT levels were more common in girls. In our study, only two of the nine patients with elevated transaminase levels were girls. This discrepancy may be attributed to the retrospective, single-center design of our study and the relatively small sample size. The authors of this study suggested that, given the high prevalence of abnormal liver enzyme levels in children with CF, liver function tests should be performed more frequently. However, they emphasized that further studies are needed to determine the optimal screening interval.

In a study conducted in Canada, 106 pediatric patients diagnosed with CF were followed for approximately 10 years [20]. Initially, 10% of these patients had clinical and laboratory evidence of liver disease; however, all had normal ultrasound findings. At the end of the study, abnormal ultrasound findings were detected in 19 patients (17.9%) (heterogeneous parenchyma in 15 patients and a nodular liver in 4 patients), but these changes were not consistent with biochemical tests. Portal HT developed in eight of these patients. Consistent with that study, abnormal ultrasound findings were detected in 13 (21.6%) patients in the present study; five patients had hepatomegaly, six had a heterogeneous granular appearance, and two had hepatosteatosis. In our study, children with elevated ALT levels (> 40 U/L) were nearly five times more likely to have abnormal liver ultrasonography findings than those with normal ALT levels (OR = 4.69). However, this association was not statistically significant. The lack of statistical significance was likely attributable to the limited number of patients who underwent liver ultrasonography, particularly the small number of patients with elevated ALT levels, which reduced the statistical power to detect a significant association. Nevertheless, the observed effect size (OR = 4.69) suggests a potentially clinically relevant association between elevated ALT levels and abnormal liver ultrasonography findings. These findings should be interpreted with caution and warrant confirmation in larger, prospective studies. Overall, our results further emphasize the heterogeneous nature of cystic fibrosis–associated hepatobiliary involvement and suggest that biochemical parameters alone may be insufficient to identify all patients with hepatobiliary abnormalities [21].

Liver enzyme fluctuations are frequent and can be caused by factors such as medications, infections, and poor nutritional status [20, 22]. Elevated liver enzyme levels outside of these periods are an important diagnostic criterion for detecting liver involvement. During these periods, transient elevations were observed in approximately half of our patients. Patients with elevated liver enzyme levels on at least two consecutive measurements obtained at different time points were considered to have hepatobiliary involvement.

In a prospective study conducted by Costa et al. [18], 62 patients with CF were evaluated between 1994 and 2009. Patients underwent clinical examinations every 2 months, biochemical tests every 6 months, and annual liver USG. The cumulative prevalence of liver disease was 11.2% (7 cases). Liver involvement was detected at a mean age of 8 years. Four patients underwent liver biopsy; one patient was found to have biliary fibrosis, two to have focal biliary cirrhosis, and one to have multilobular cirrhosis. All patients with hepatobiliary involvement were started on UDCA. In our study, hepatobiliary involvement was detected in 18 patients (30.0%), representing a higher prevalence than that reported by Costa et al. [18]. In addition to that, only one patient underwent liver biopsy and the biopsy findings were consistent with cirrhosis in the present study. This difference may be attributable to differences in study design, patient characteristics, diagnostic criteria, and the relatively small sample size. Consistent with the findings of Costa et al. [18], UDCA was initiated all patients with hepatobiliary involvement in our study.

Liver disease was generally detected during the first decade of life in patients with CF, and the authors emphasized the role of ultrasonography in the early detection of hepatobiliary involvement [18]. Similarly, eight patients in our study had abnormal liver ultrasonography despite normal serum transaminase levels. This finding underscores the complementary value of liver ultrasonography and serum transaminase assessment for the early detection and evaluation of CF-related liver disease.

In the study conducted by Scott-Jupp et al. [23], it was observed that the majority of patients with hepatobiliary involvement were diagnosed before the age of 10 years. Consistent with previous studies [18, 23, 24], 53.8% (10/18) of the patients with hepatobiliary involvement in our study were younger than 10 years of age.

Cheng et al. [25] reported a response rate of over 70% to UDCA treatment. In our cohort, the response rate was approximately 83%, consistent with their findings. UDCA is considered the standard treatment for the management of CFALD. However, portal HT and the need for liver transplantation are serious complications in patients with advanced liver disease. Early referral for liver transplantation evaluation is recommended for patients with ultrasonographic evidence of portal HT (e.g., splenomegaly or collateral vessel formation) [26]. Although only one patient in our study met these criteria, the patient was referred to a liver transplantation center. The frequency of these complications may increase with long-term follow-up. Consistent with the study by Cheng et al. [25], UDCA was initiated in all patients with hepatobiliary involvement in our study.

### Strengths and limitations

This study has several strengths. It provides real-world data on hepatobiliary involvement in a well-defined cohort of pediatric patients with cystic fibrosis from a tertiary referral center. Clinical findings, liver function tests, and liver ultrasonography were evaluated together, allowing for a comprehensive assessment of hepatobiliary involvement. Furthermore, patients with transient liver enzyme elevations related to infections, medications, or nutritional support were excluded, thereby reducing potential misclassification.

This study has several limitations. First, its retrospective, single-center design and relatively small sample size may have reduced the precision of the prevalence estimates, limited the robustness of the findings, and restricted their generalizability to the broader pediatric cystic fibrosis population. Second, the inclusion of only patients with available liver-related investigations and laboratory data, together with the exclusion of patients with missing evaluations, may have introduced selection bias by preferentially including patients with more regular follow-up and better healthcare engagement. Consequently, the estimated prevalence of hepatobiliary involvement may not accurately reflect the true prevalence in the overall pediatric cystic fibrosis population. Third, liver ultrasonography was not performed in 13 patients. These patients were excluded from analyses involving ultrasonographic findings, which may have introduced selection bias and reduced the statistical power of those analyses. In addition, ultrasonographic examinations were performed by different radiologists, and interobserver variability may have influenced the imaging findings. Furthermore, the retrospective design relied on the accuracy and completeness of medical records and limited the standardization of clinical, laboratory, and imaging assessments. Although patients with transient liver enzyme elevations related to infections, medications, or nutritional support were excluded, some degree of outcome misclassification cannot be entirely ruled out. Finally, the relatively short follow-up period and the absence of complete long-term follow-up data limited our ability to evaluate the long-term clinical significance and progression of hepatobiliary involvement. Therefore, our findings should be interpreted with caution, and larger, prospective, multicenter studies with standardized imaging protocols and longer follow-up are needed to confirm these findings.

## Conclusions

As a result, 13 of our patients did not come for follow-up visits, so US could not be performed on these patients. Ultrasonography findings were abnormal in 8 patients with normal transaminase levels. However, hepatobiliary involvement was detected in 18 (30.0%) patients in the current study. The presence of abnormal US findings without affecting liver enzyme levels demonstrates the importance of US in screening patients with CF for liver disease. Normal liver function tests do not necessarily mean the liver is not affected.

Our findings highlight the importance of regular monitoring for hepatobiliary involvement in children with CF. However, recommendations regarding screening intervals should be based on established clinical guidelines rather than on the findings of this study alone. Further prospective studies are needed to determine the optimal screening approach. 

## Data Availability

No datasets were generated or analysed during the current study.

## Abbreviations

CF: Cystic fibrosis
CFTR gene: Cystic Fibrosis Transmembrane Regulator gene
CFALD: Cystic Fibrosis-Associated Liver Disease
HT: Hypertension
US: Ultrasound
UDCA: Ursodeoxycholic acid
